# A Case of Cryptococcal Hepatitis in an HIV Patient with a Negative Serum Cryptococcal Antigen

**DOI:** 10.7759/cureus.6496

**Published:** 2019-12-28

**Authors:** Elias Estifan, Ian Laxina, Sami Adib, Jin S Suh, Walid Baddoura

**Affiliations:** 1 Internal Medicine, St. Joseph's University Medical Center, Paterson, USA; 2 Gastroenterology, St. Joseph's University Medical Center, Paterson, USA; 3 Infectious Disease, St. Joseph's University Medical Center, Paterson, USA

**Keywords:** cryptococcus neoformans, cryptococcal hepatitis, hiv, hiv related disease

## Abstract

Infectious *Cryptococcus neoformans* occurs primarily in immunocompromised patients. The primary organ affected is the lungs, but the infection of the central nervous system (CNS) is also be seen. Disseminated cryptococcosis can involve any organ in the body. However, hepatic involvement is rare. Here we discuss a case of cryptococcal hepatitis in a patient who presented with persistently elevated liver enzymes. A 56-year-old Ecuadorian female with no known past medical history presented with fever, abdominal pain, nausea, unintentional weight loss, and diarrhea for two months. Her liver function tests (LFTs) revealed elevated aspartate aminotransferase (AST: 415 U/L), elevated alanine aminotransferase (ALT: 201 U/L), elevated alkaline phosphatase (ALP: 763 U/L), but normal total bilirubin (0.9 mg/dl). Her HIV antigen screening was reactive, and the absolute cluster of differentiation 4 (CD4) helper count was 22 cell/µL. Over the course of her hospital stay, the patient's liver enzymes continued to trend upward, with negative Histoplasma antibodies and negative serum cryptococcal antigen titers. During the second week of hospitalization, her liver enzymes continued to rise with an ALP of 4046 U/L, AST of 436 U/L, and ALT of 276 U/L. With a persistent elevation of the liver enzymes without any definitive cause, an ultrasound-guided biopsy was performed. Pathology revealed cryptococcal hepatitis, and the patient was started on a 15-day course of amphotericin B with an eight-week course of fluconazole 400 mg with LFTs nearly normalizing at six weeks. This case demonstrates an unusual manifestation of cryptococcosis. Our patient did not present with the typical cryptococcal pulmonary or central nervous system infection. Additionally, our patient's serum cryptococcal antigen titers were negative, but biopsy results revealed cryptococcal hepatitis, despite a very high sensitivity and specificity of the serum cryptococcal antigen test. This case demonstrates the importance of maintaining a broad differential, specifically in immunocompromised patients.

## Introduction

*Cryptococcus neoformans* occurs primarily in immunocompromised patients such as those who have contracted the human immunodeficiency virus (HIV), organ transplant recipients, and patients on long-term corticosteroid. While lungs are the primarily affected organ, the central nervous system (CNS) and the skin are also commonly affected. Disseminated cryptococcosis can involve almost every organ in the body; however, isolated hepatic involvement is very rare. We present here a rare case of cryptococcal hepatitis in a patient who presented with a negative serum cryptococcal antigen [1].

## Case presentation

A 56-year-old Ecuadorian female with no known past medical history was seen for fever, abdominal pain, nausea, and diarrheas. She had been experiencing intermittent right upper quadrant pain, which was non-radiating and unrelated to food ingestion. She also complained of associated fever, nausea, and watery, non-bloody diarrhea for two months' duration. Her review of systems was negative except for an unintentional weight loss 15 pounds with a decrease in her appetite. On physical examination, she was tachycardic with a heart rate of 110 bpm. The abdomen was soft, tender to palpation at the right upper quadrant. Her liver function tests (LFTs) revealed elevated aspartate aminotransferase (AST: 415 U/L), elevated alanine aminotransferase (ALT: 201 U/L), elevated alkaline phosphatase (ALP: 763 U/L), low albumin (2.6 g/dl), normal total bilirubin (0.9 mg/dl), prothrombin time of 15.1 seconds, international normalized ratio (1.2), and partial thromboplastin time (36.0 seconds). Her comprehensive metabolic panel is presented in Table 1. Complete blood count revealed white blood cells of 3.0 x 10^3/mm^3 with differential of 96% segments, 0% bands, 2% lymphocytes, 2% monocytes, 0% eosinophils, and 0% basophils; hemoglobin of 10.8 g/dL; and platelets of 129 K/mm^3. Additional laboratory showed reactive HIV antigen screen, absolute cluster of differentiation 4 (CD4) helper count at 22 cells/µL with a viral load of 72,750 copies/mL, negative blood culture x2, negative stool culture, negative ova, and parasites, negative Clostridium difficile toxin/antigen, and negative cryptosporidium feces antigen. She was deemed a newly diagnosed HIV patient without any significant risk factors except for a blood transfusion in her home country five years before the presentation. She also denied being sexually active for the last four years. She denied any history of alcohol use, smoking, or illicit drug abuse. She had moved from Ecuador to the US at the age of 50 years. She was a housewife and had never worked in a factory or had exposure to chemicals.

**Table 1:** Comprehensive metabolic panel and additional laboratory test results of the patient BUN: blood urea nitrogen; ALP: alkaline phosphatase; AST: aspartate aminotransferase; ALT: alanine aminotransferase; GGT: gamma-glutamyl transpeptidase; HIV: human immunodeficiency virus; CD4: cluster of differentiation 4

	At admission	Week 1	Week 2	Week 3	Week 4	Week 5	Week 6
Sodium (meq/L)	129	140	135	141	148	139	129
Potassium (meq/L)	3.5	3.8	3.9	3.8	4.1	3.7	3.9
Chloride (meq/L)	95	113	107	109	114	110	97
Bicarbonate (meq/L)	18	21	18	20	18	20	20
BUN (mg/dl)	8	9	9	20	18	14	10
Creatinine (mg/dl)	0.56	0.35	0.28	0.87	1.11	0.47	0.33
Bilirubin Total (mg/dl)	0.9	3.7	4.0	1.5	1.0	0.6	0.4
Bilirubin direct (mg/dl)	0.3	2.5					
Protein total (g/dl)	7.5	4.5	5.5	7.8	8.7	7.3	6.2
Albumin (g/dl)	2.6	1.7	2.1	3.2	3.6	3.1	3.1
ALP (U/L)	763	1,091	4,046	2,554	1,207	807	120
AST (U/L)	415	319	436	143	144	84	29
ALT (U/L)	201	108	276	125	109	105	33
GGT (U/L)	1,147						
Lipase (U/L)	38						
HIV	Reactive						
CD4 (mcL)	22						
HIV viral load (copies/mL)	72,750						

**Figure 1:**
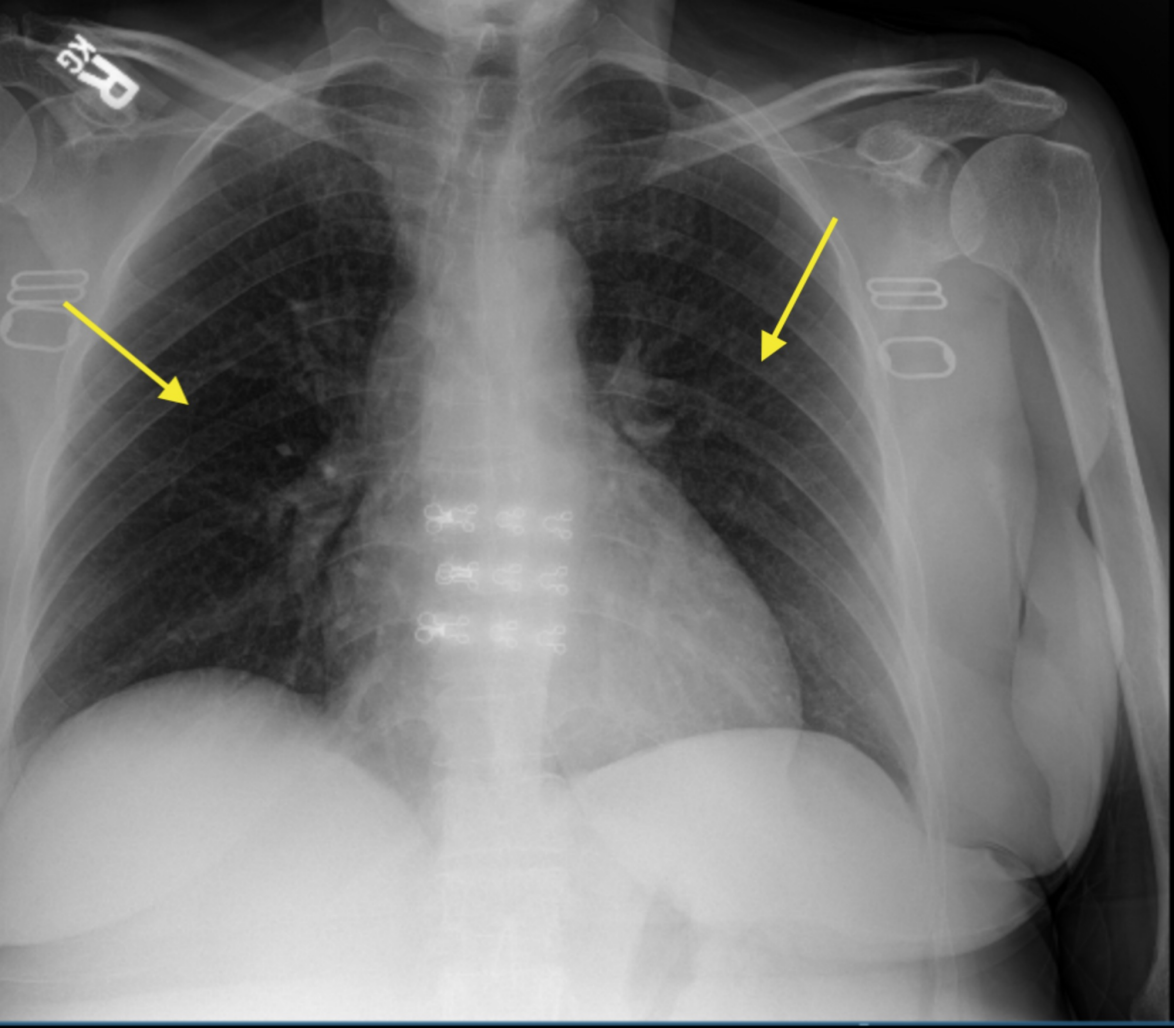
Chest X-ray showing no significant findings (yellow arrows)

**Figure 2:**
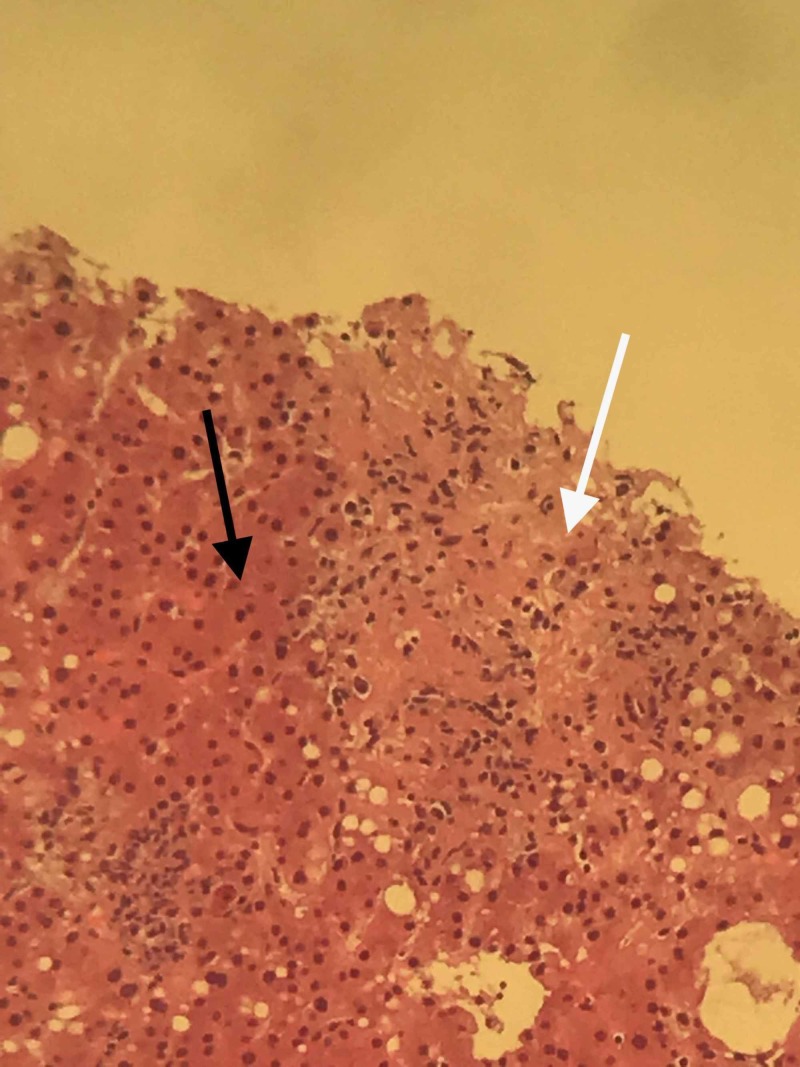
Pathology image of the patient The image shows a normal hepatic tissue (black arrow) with poor ill-defined granuloma (white arrow)

**Figure 3:**
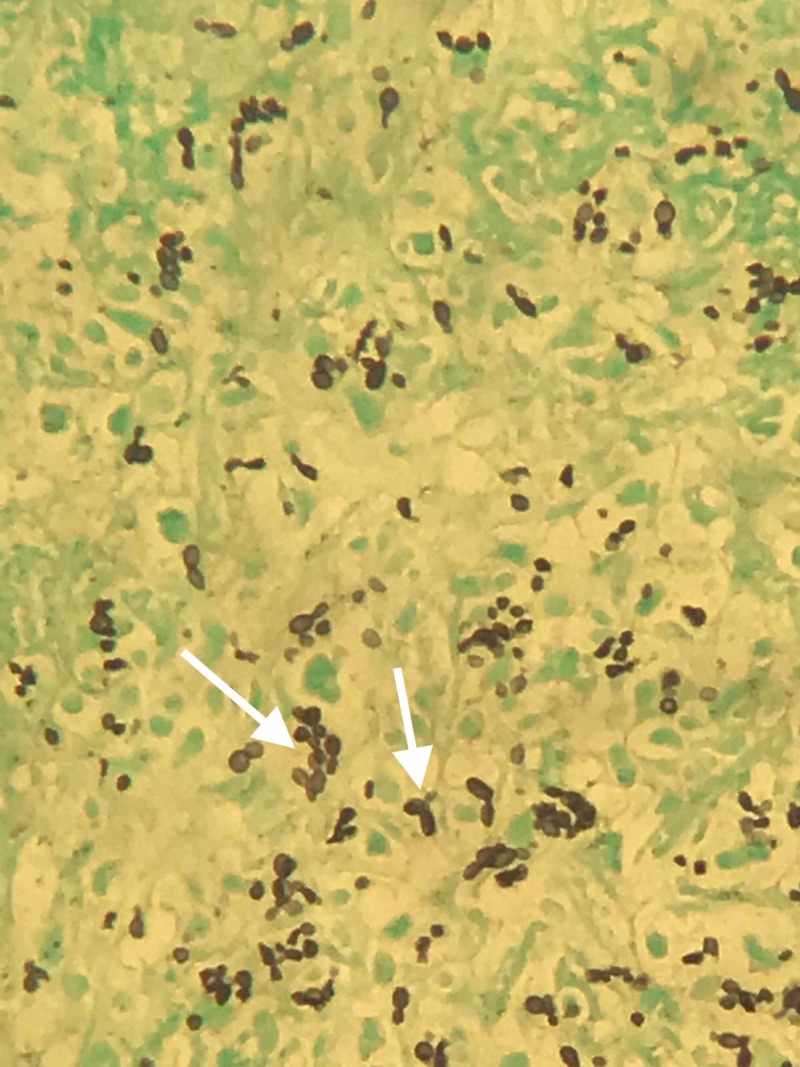
Pathology image of Grocott’s Methenamine Silver (GMS) stain The image shows multiple yeast spore noted with different sizes (specific for *cryptococcus*) and noted narrow budding (white arrow)

A chest X-ray showed no significant findings (Figure 1). Right upper quadrant ultrasound revealed an enlarged liver at 19.6 cm in length and diffusely echogenic consistent with fatty infiltration. Due to the patient’s HIV status and unimproved symptoms, she was started on azithromycin for mycobacterium avium complex (MAC) prophylaxis and trimethoprim-sulfamethoxazole for empiric treatment of suspected *Pneumocystis Jiroveci* Pneumonia (PJP). Meanwhile, the patient's liver enzyme continued to trend up and a further workup revealed negative viral hepatitis A, B, C, negative antinuclear antibody (ANA), mildly elevated anti-smooth muscle antibody (ASMA) at 30 units, negative ceruloplasmin (34.5 mg/dL), negative Histoplasma antibody, negative cytomegalovirus (CMV) IgM Ab (<30 AU/mL), and negative serum cryptococcal antigen titers. In the second week of hospitalization, her liver enzymes continued to worsen with ALP of 4046 U/L, AST of 407 U/L, and ALT of 276 U/L without any definitive cause. An ultrasound-guided biopsy was performed, and pathology revealed cryptococcal hepatitis with negative acid-fast bacilli and cytomegaloviral stain (Figure 2). On Grocott’s methenamine silver stains, the granulomas showed multiple yeast forms consistent with *Cryptococcus* (Figure 3). The patient was then started on amphotericin B for 15 days and fluconazole 400 mg for eight weeks with near normalizing in her liver function at the six-week mark.

## Discussion

*Cryptococcus* is an encapsulated yeast-like fungus. There are two variants of *Cryptococcus*: *neoformans* and *gatti*. *Cryptococcus neoformans* variety is a well-known disease in immunosuppressed individuals. The *gatti* form occurs in healthy individuals. It is usually acquired by inhalation of aerosolized infectious particles and found in soils contaminated with avian excreta. The spectrum of disease caused by *Cryptococcus* species consists predominantly of meningoencephalitis and pneumonia, but skin and soft tissue infections also occur; in fact, cryptococcosis can affect any tissue or organ. Patients with hematologic malignancies, advanced HIV infection, and CD4+ T lymphocyte counts of <200 cells/µl, recipients of solid organ transplants, and patients on glucocorticoid therapy have a higher risk for cryptococcal infection. Although serology evidence of cryptococcal infection is common among immunocompetent individuals, the cryptococcal disease is relatively rare in the absence of impaired immunity. However, with the advent of effective antiretroviral treatment, the incidence of acquired immune deficiency syndrome (AIDS)-related cryptococcosis has been sharply reduced among treated individuals. Therefore, most cases of cryptococcosis now occur in resource-limited regions of the world [2-5].

In HIV infection, the liver and biliary tract could be involved with infections mostly mycobacteria and cytomegalovirus, acalculous cholecystitis, sclerosing cholangitis, and neoplasms, mainly Kaposi’s sarcoma. Also, the consumption of HIV-related medications, including sulfa drugs, pentamidine, and ketoconazole can result in abnormalities in LFTs [6]. Several patients with AIDS and cryptococcosis have extra-pulmonary/neural disease. In 19% of these patients, the organism can be found in the liver by histology or culture. Positive cultures are less common in liver tissue (12%) than in blood (32%) or bone marrow (50%). The levels of liver enzymes and bilirubin may be elevated, but the liver involvement is likely to remain asymptomatic [7,8]. In two studies of HIV patients with abnormal liver function tests who underwent a liver biopsy, the first study, which included 635 patients, showed cytomegalovirus count of 4%, mycobacterium avium-intracellulare count of 11%, *Cryptococcus neoformans* count of 2% and Kaposi sarcoma count of 8.6% [6]. In the second study of 46 patients, cryptococcosis was positive in 13% of all the patients, with one case combining *Cryptococcus* and mycobacterium tuberculosis in one patient [9]. Interestingly, one case in the literature reported founding a cryptococcal liver abscess in a patient with myelodysplastic syndrome status post-splenectomy [10].

This case demonstrates the unusual manifestation of cryptococcosis. Interestingly our patient did not present with typical cryptococcal pulmonary infection or any sign of neurological involvement. However, her liver enzyme continued to trend up, and serum cryptococcal antigen titers were negative. Liver biopsy was done, which exhibited underlying cryptococcal hepatitis despite a very high sensitivity and specificity of the serum cryptococcal antigen test. Tanner et al. found that the sensitivity of serum cryptococcal antigen ranged from 83% to 97% and the specificity from 93% to 100%; false-positive rates ranged from 0 to 5% [11]. In other studies, cryptococcal meningitis is diagnosed with cryptococcal cerebrospinal fluid (CSF) antigen and had an overall sensitivity of 94.1 %, followed by the serum cryptococcal antigen of 93.6% [12].

## Conclusions

The spectrum of cryptococcosis in HIV-infected patients is so varied and has changed so much since the advent of antiretroviral therapy that a distinction between HIV-related and HIV-unrelated cryptococcosis is no longer pertinent. This case demonstrates the importance of maintaining a broad differential, specifically in immunocompromised patients.
